# Supplementary material for: Single-shot polarimetry of vector beams by supervised learning
Source: Nat Commun. 2023 Apr 1;14:1831. doi: 10.1038/s41467-023-37474-0 (PMC10067938; doi:10.1038/s41467-023-37474-0)
Supplement: Supplementary file 1 — Supplementary Information [file 41467_2023_37474_MOESM1_ESM.pdf]

Supplementary Information for:
Single-shot polarimetry of vector beams by supervised learning

Davide Pierangeli *[1,2,3] and Claudio Conti[2,1,3]

[1] *Institute for Complex Systems - National Research Council (ISC-CNR), 00185 Rome, Italy*
[2] *Physics Department, Sapienza University of Rome, 00185 Rome, Italy*
[3] *Research Center Enrico Fermi (CREF), 00184 Rome, Italy*

* Corresponding Author: Davide Pierangeli. davide.pierangeli@roma1.infn.it

Supplementary Note 1. Generation of multi-parted vector beam by spatial light modulation

To apply a phase-only spatial light modulator (SLM) as a programmable generator of light beams encoding multiple polarizations, we first characterize the effect of our liquid-crystal on silicon (LCS) SLM device (Hamamatsu X13138) on the reflected zero-order polarization, for a linearly polarized input wave forming an angle θ with the plane of the experiment (horizontal, H) and with the major axis of the active display (x-axis). By varying both θ and the constant grayscale value displayed on the SLM, we measure the Jones matrix of the SLM as a function of the programmed phase delay ϕ. We found that the SLM input and output components of the optical field, respectively E_x^{in}, E_y^{in} and E_x^{slm}, E_y^{slm}, are approximately related by the expression

$$\begin{pmatrix} E_x^{slm} \\ E_y^{slm} \end{pmatrix} = \begin{pmatrix} e^{i\phi} & 0 \\ 0 & i \end{pmatrix} \begin{pmatrix} E_x^{in} \\ E_y^{in} \end{pmatrix}. \tag{1}$$

For a diagonal (D) input SOP we get left (L) and right (R) circular outgoing polarization, respectively for $\phi = 0$ and $\phi = \pi$. Diagonal polarization at the SLM input is used throughout the experiments. To generate an arbitrary state of polarization (SOP) by varying ϕ, we use a quarter-wave plate (QWP) and a half-wave plate (HWP) after the

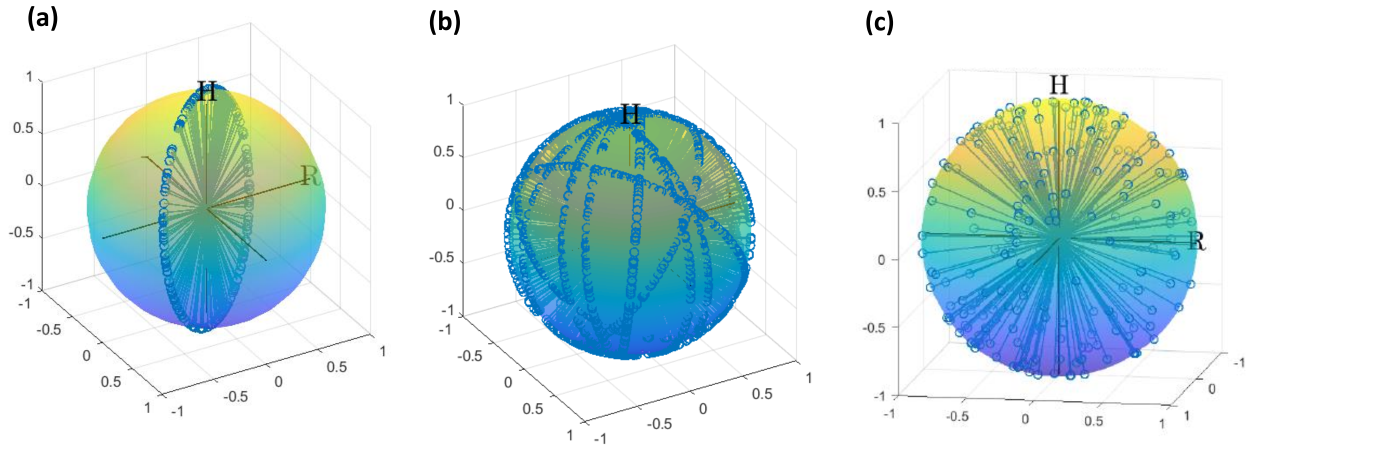

Supplementary Figure 1. Generating arbitrary polarization states with a phase-only SLM and rotating waveplates. (a) Varying ϕ generates a set of states on a circle on the Poincaré-Bloch (PB) sphere. (b) The azimuthal and polar angles of the circle can be varied through α and β. (c) The entire PB surface can be sampled with the parameter set $\{\phi, \alpha, \beta\}$. The shown points are drawn randomly from a uniform distribution.

SLM (see Fig. 2a of the main text), with their fast axes forming an angle α and β with respect to the x-axis. The components of the generated optical field read as

$$E_x^{out} = \left[\cos(2\beta)[\cos^2(\alpha) + i\sin^2(\alpha)] + \sin(2\beta)(1-i)\sin(\alpha)\cos(\alpha)\right] E_x^{in}\exp(i\phi)+ \\ + \left[\cos(2\beta)(1-i)\cos(\alpha)\sin(\alpha) + \sin(2\beta)[\sin^2(\alpha) + i\cos^2(\alpha)]\right] E_y^{in} \tag{2}$$

$$E_y^{out} = \left[\sin(2\beta)[\cos^2(\alpha) + i\sin^2(\alpha)] - \cos(2\beta)(1-i)\sin(\alpha)\cos(\alpha)\right] E_x^{in}\exp(i\phi)+ \\ + \left[\sin(2\beta)(1-i)\cos(\alpha)\sin(\alpha) - \cos(2\beta)[\sin^2(\alpha) + i\cos^2(\alpha)]\right] E_y^{in} \tag{3}$$

For a given couple (α, β), the SOP spans a circle on the PB sphere surface as we vary ϕ in the range $[0, 2\pi]$, as shown in Supplementary Fig. 1(a). By means of the three generator's parameters ϕ, α, and β, we fully control the generated polarization. A set of randomly-generated SOP is shown on the Poincaré-Bloch (PB) sphere in Supplementary Fig. 1(c).

Light encoding multiple SOP is generated by dividing the SLM in D squared macro-pixels with D independent phase values ϕ^j (Supplementary Fig. 2(a)), for fixed α and β values. To characterize the generator, the polarization of a set of randomly-generated vector beams is analyzed using the rotating quarter-waveplate method [1]. An example of a beam with $D = 4$ and its projective analysis is reported in Supplementary Fig. 2. The intensity signal varying the analyzer quarter waveplate is obtained by averaging over an image region of interest (ROI). The ROI is selected by post-processing each image, manually or by using a search algorithm. The Stokes parameters are obtained from the intensity analysis function [1]. For example, the intensity curve in Fig. S2(h) gives $(S_1, S_2, S_3, \nu) = (-0.48, 0.62, -0.53, 0.94)$. The average distance with respect to the expected SOP is $d = 0.07 \pm 0.02$, which also includes the uncertainty of the polarization analyzer.

Supplementary Note 2. Model of the single-shot polarization measurement

The key ingredient of single-shot polarimetry is the mapping of the polarization state into a set of distinct observable intensities by using a physical component of the setup. In our setting, this operation is performed by a disordered medium through linear optical propagation. To model the transformation performed by the scattering medium on the input SOP, we consider the optical interaction in the framework of the optical transmission matrix (TM). The TM models field transmission through a linear optical system at the mesoscopic level and, in particular, multiple light scattering between D input modes and C output modes [2]. For a vectorial field, the vectorial TM complex coefficients t_{mn}^{kl} connect the input field E_n^k, with polarization state k in the n-th spatial input mode, to the output field E_m^l, with polarization state l in the m-th output mode [3]. Specifically, we have

$$\begin{pmatrix} E_m^x \\ E_m^y \end{pmatrix} = \sum_n \begin{pmatrix} t_{mn}^{xx} & t_{mn}^{xy} \\ t_{mn}^{yx} & t_{mn}^{yy} \end{pmatrix} \begin{pmatrix} E_n^x \\ E_n^y \end{pmatrix}. \tag{4}$$

Complete information on the vectorial TM allows predicting the output polarization at any point from a given phase profile at the input. When a thick disordered medium is placed between a SLM and a camera, the $2D \times 2C$ elements t_{mn}^{kl} are uncorrelated random complex numbers. The lack of correlations among the TM elements gives the depolarization effect that characterizes optical propagation of uniformly-polarized beams in thick multiple-scattering media [4]. In this case, every spatial mode in the transmission plane has a random polarization. On the contrary, when the medium thickness is much smaller than the transport mean free path, the scattering process can conserve the incoming polarization, i.e., input wavevectors are rotated without polarization scrambling [5]. The intensity on the m-th output channels writes as

$$I = |E^x + E^y|^2 = |\sum_n t_n^{xx} E^x + \sum_n t_n^{xy} E^y + \sum_n t_n^{yx} E^x + \sum_n t_n^{yy} E^y|^2 \tag{5}$$

where the m index has been omitted for clarity, and $1 < n < D$. For an isotropic disordered medium the interaction is symmetric with respect to the reference axes, which implies $t_n^{xy} = t_n^{yx}$. At the leading orders, we have

$$I \approx |\sum_n t_n^{xx} E^x|^2 + |\sum_n t_n^{yy} E^y|^2 + |\sum_n t_n^{xy}(E_n^y + E_n^x)|^2. \tag{6}$$

We analyze Eq. (6) starting for simplicity from the case of a single SOP at the input. For $D = 1$, Eq. (6) reduces to $I \approx |t^{xx}E^x|^2 + |t^{yy}E^y|^2 + t^{xy}\bar{t}^{xy}|E^y + E^x|^2$. Zero coupling between input polarization and the spatial amplitude means

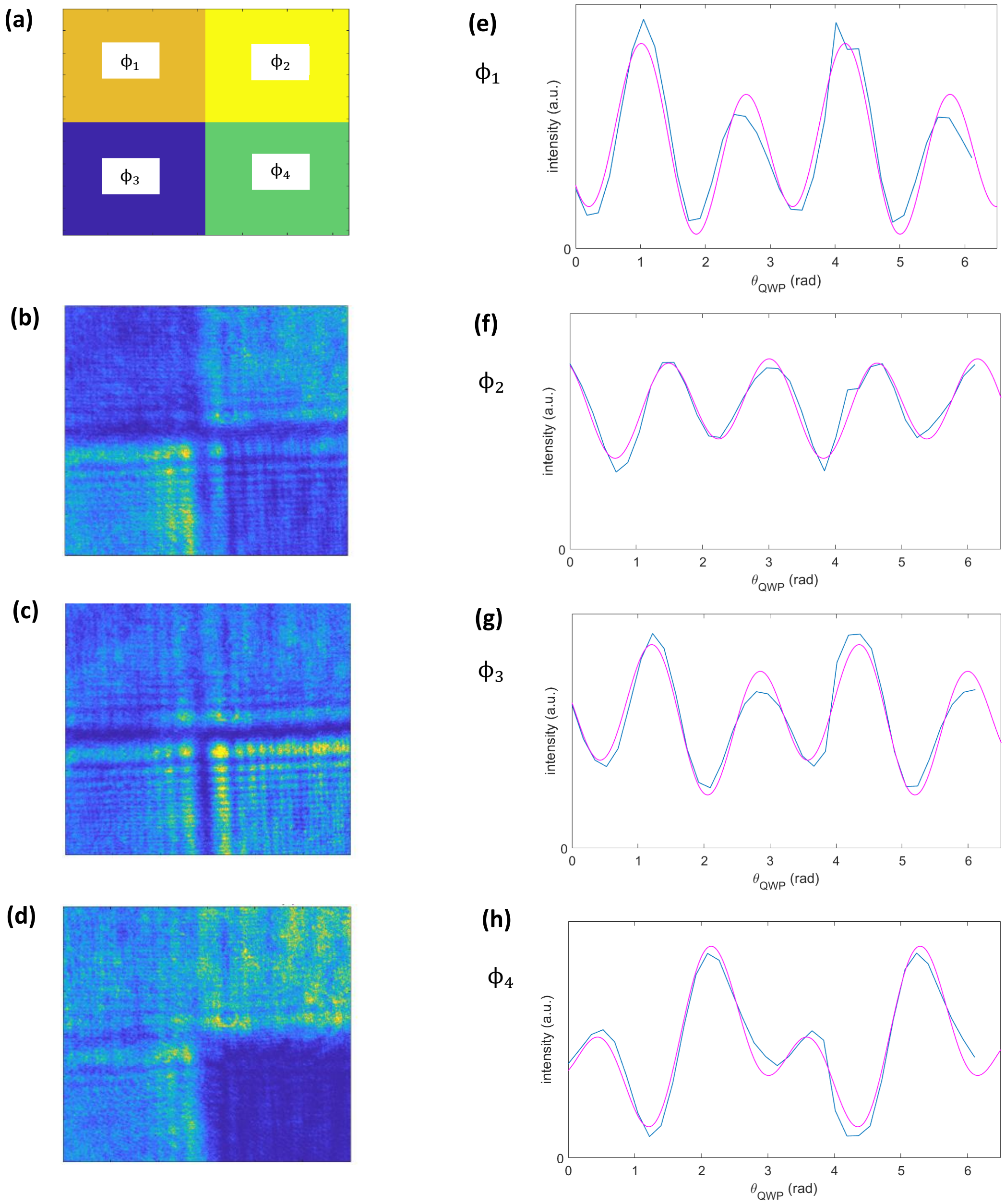

Supplementary Figure 2. Conventional polarimetry of partitioned vector beams with $D = 4$. (a) Phase mask on the SLM with four blocks of pixels (input modes) having four different phases ϕ^j. (b-d) Examples of intensity images when projecting the vector beam through a LP and a QWP at various angles θ_{QWP}. (e-h) Measurements of the polarization in each mode through the rotating quarter-waveplate method [1]. Shown is the averaged intensity varying the QWP angle (blue line), along with the best fit (magenta line) providing the Stokes parameters.

$t^{xy} = 0$ and $t^{xx} = t^{yy}$, i.e., absence of polarization rotation. In this case, the output field at position m is related to the input field through a random complex coefficient, and thus no method can extract the input polarization from the intensity data. Non-zero coupling is a necessary condition of our single-shot method for $D = 1$. A measurement of the t^{kl}_{mn} coefficients is reported in Supplementary Fig. 4, which confirms the mechanism by showing the presence of small coupling ($t^{xy} \ll 1$). If we suppose the zero-coupling condition in the general case of an input beam composed of

several SOP in D spatial modes, the output field in a given spatial point reads as $E = \sum_{n=1}^{D} [t_n^{xx} E_n^x + t_n^{yy} E_n^y]$, which is a coherent superposition of all the input SOP. The intensity on the m-th point of the detector plane would be

$$I_m \approx \sum_{p,q} t_p^{xx} \bar{t}_q^{xx} E_p^x E_q^x + \sum_{p,q} t_p^{yy} \bar{t}_q^{yy} E_p^y E_q^y + \sum_{p,q} \left[t_p^{xx} \bar{t}_q^{yy} + \bar{t}_q^{xx} t_p^{yy} \right] E_p^x E_q^y \tag{7}$$

with $p = 1, ..., D$, and $q = 1, ..., D$. Eq. (8) expresses the transformation performed by the scattering medium on the partitioned vector beam in the eventual absence of polarization-spatial coupling. This approximate relation between the local scattered intensity and the local input polarization illustrates how coupling induced by the scatterer is not mandatory for the operation of our method in the vector case. The partitioning of the input beam makes the output intensity distribution depend on the multiple SOP. In Supplementary Note 3, by measuring the scattered polarization and the vectorial transmission matrix, we show that small coupling conditions apply in our experiments. This guarantees the operating principle of our single-shot polarimeter.

For single-shot polarimetry, we select a set of $4M$ channels among the C available output modes. The signal x_i we collect in each channel approximately reads as

$$x_i = \mathrm{f}\left[I_i(E_p^x, E_p^y) \right], \tag{8}$$

where f is a nonlinear response function associated with the camera sensor. We work in unsaturated conditions, i.e., the camera signal is proportional to the optical intensity $x_i = I_i$, but a saturable nonlinear function ($\mathrm{f}[u] = u/(1+u)$) can be obtained by increasing the exposure time [6]. Considering the Stokes parameters definition

$$\begin{aligned} S_0^p &= |E_p^x|^2 + |E_p^y|^2, \\ S_1^p &= |E_p^x|^2 - |E_p^y|^2, \\ S_2^p &= 2|E_p^x||E_p^y|\cos(\delta_p), \\ S_2^p &= 2|E_p^x||E_p^y|\sin(\delta_p), \end{aligned} \tag{9}$$

with $\delta_P = E_p^y - E_p^x$, we obtain that the intensity on the i-th channel depends on the entire input Stokes vector $|\mathbf{s}\rangle$, $I_i = I_i(S_1^1, ..., S_0^1 ..., S_1^D, ..., S_0^D)$. Therefore, the collected signal can be expressed via the matrix equation

$$|x\rangle = \hat{T}|\mathbf{s}\rangle, \tag{10}$$

where the operator $\hat{T}$ that performs the transformation has size $4D \times 4M$. Its coefficients T_{mn}^{kl} can be recast in terms of the vector TM coefficients t_{mn}^{kl}. Once a calibration matrix $\hat{\beta}$ has been determined, the intensity signal $|x\rangle$ allows single-shot measurement of the input multiple SOP as

$$|\mathbf{s_o}\rangle = \hat{\beta}|x\rangle = (\hat{\beta}\hat{T})|\mathbf{s}\rangle, \tag{11}$$

which generalizes Eq.(1) of the main text to the vector beam case. For a single SOP, we use M channels for each Stokes parameter and the measurement outcome is

$$S_i = \sum_{k=1}^{M} \beta_k x_k, \tag{12}$$

which is the result of a linear classifier trained via the calibration vector $\hat{\beta}$.

Supplementary Note 3. Measurement of the vectorial transmission matrix

In this supplementary note, we report measurements of the vectorial transmission matrix of the scattering medium used to perform the mapping of the polarizations into a spatial intensity distribution. We first verify that the scattering process does not destruct an incoming SOP. To show that the employed ground glass diffuser maintains most of the input polarization, we analyze the polarization of the intensity speckle pattern produced by a generic elliptical polarization ($D = 1$). As reported in Supplementary Fig. 3(b), different spatial points have a strongly-correlated polarization, indicating there is no mixing between the field components of a given input mode. This is confirmed through measurements of the Stokes parameters with a conventional polarimeter (see Methods). The average distance between the incoming and the transmitted SOP is $d = 0.05 \pm 0.01$ and the degree of polarization

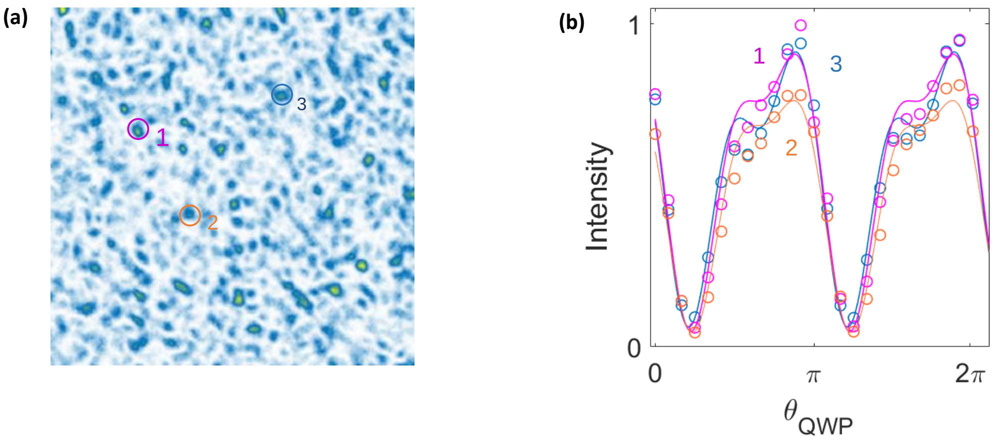

Supplementary Figure 3. (a) Intensity distribution transmitted by the scattering medium for a generic uniform polarization. (b) The polarization of distinct speckle grains is measured by varying the QWP angle of the projective analyzer. The speckle pattern has small polarization fluctuations, which indicates that the input SOP is partially conserved by the scattering process and evidences the small coupling generated by the scatterer.

of the scattered field is $\nu = 0.97$. This polarization-maintaining effect does not occur when using a thick scattering sample, as we observe when using a sample made of teflon with 0.2mm thickness ($\nu = 0.1$).

The vectorial TM is measured in both the H-V basis (x-y basis) and R-L basis (circular basis) by fixing the input and output polarization and performing independent measurements for the same realization of disorder (sample position). For a selected configuration of the polarizer and analyzer, we randomly vary the phase configuration of the D input SLM modes. For each of the N_{samples} input phase masks, we acquire the transmitted intensity on C camera modes. The TM is reconstructed from intensity data using a phase retrieval algorithm [7]. A typical result for $D = 9$ and $C = 1000$ is reported in Supplementary Fig. 4. We find that the non-diagonal sub-matrices have elements with amplitude of the order of $10^{-}3$, which indicates that the rotation of the input linear SOP is small. Diagonal elements have large amplitudes with values close to each other, i.e., $|t^{xx}_{nm}| \approx |t^{yy}_{nm}|$, in agreement with the observed strong speckle contrast (Supplementary Fig. 3(a)). This is confirmed by the cross-correlation matrix between the transmission sub-matrices (see Ref. [5]), as shown in Supplementary Fig. 5. The correlation C_{xxyy} is reported in Fig. S5(a), and the peak above the constant features indicates the presence of strongly-correlated elements within diagonal sub-matrices. Only spurious correlations are evident between off-diagonal sub-matrices. This confirms that the optical transformation performed by our scattering medium does not disrupt the input polarization. Similar results are observed measuring the vectorial TM on the R-L basis and varying the number of input and output modes.

Supplementary Note 4. Calibration of the single-shot polarimeter

In this section, we report additional material on the calibration of the experimental setup for single-shot polarimetry. In Supplementary Fig. 6, we show an example of a random selection of channels within the camera modes ($M = 800$ for ease of visualization). We indicate with m_x and m_y the index of the m-th output mode on the camera plane. The intensities collected by using this channel set are reported in Supplementary Fig. 6(b) for a partitioned vector beam with $D = 4$. These intensity values constitute part of the vector $|x\rangle$ collected in the single-shot detection.

An example of the calibration matrix used for single-shot polarimetry of vector beam with $D = 4$ is reported in Supplementary Fig. 7. The matrix is obtained through a training phase with $N_{\text{train}} = 7200$ samples. It is interesting to investigate the distribution of the obtained β_i^j. As shown in Supplementary Fig. 7(b), we found a bell-shaped distribution with zero mean.

We note that a different single-shot measurement can also be performed on-demand once the respective calibration matrix is available. For example, two vector beams with two different wavelengths can be characterized in alternating mode. Recalibration of the experimental setting is only necessary when the optical mapping performed by the scattering medium changes considerably due to practical effects. In our setup, we found that good performance is maintained for more than one hour.

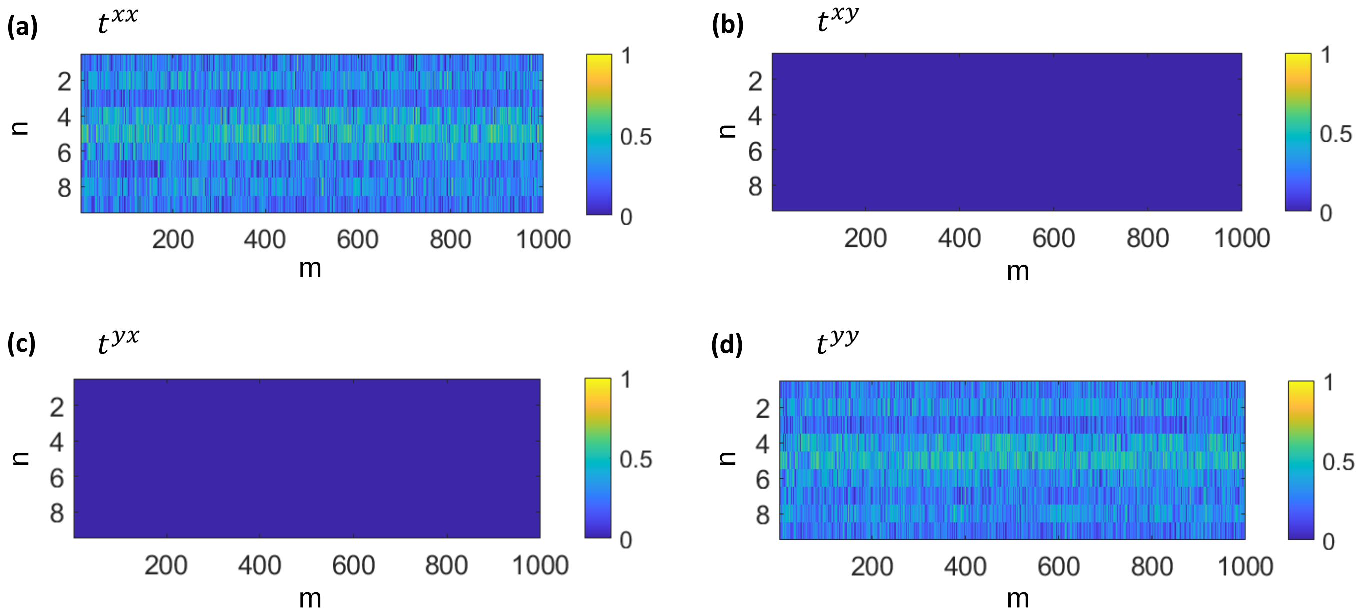

Supplementary Figure 4. Amplitude of the vectorial transmission matrix $|t_{mn}^{kl}|$ measured in the x-y basis for $D = 9$ input and $C = 1000$ output modes. Panels (a)-(d) shows the four diagonal and non-diagonal sub-matrices t^{xx}, t^{xy}, t^{yx}, t^{yy}, respectively, normalized on the same scale (cfr. Eq(1)).

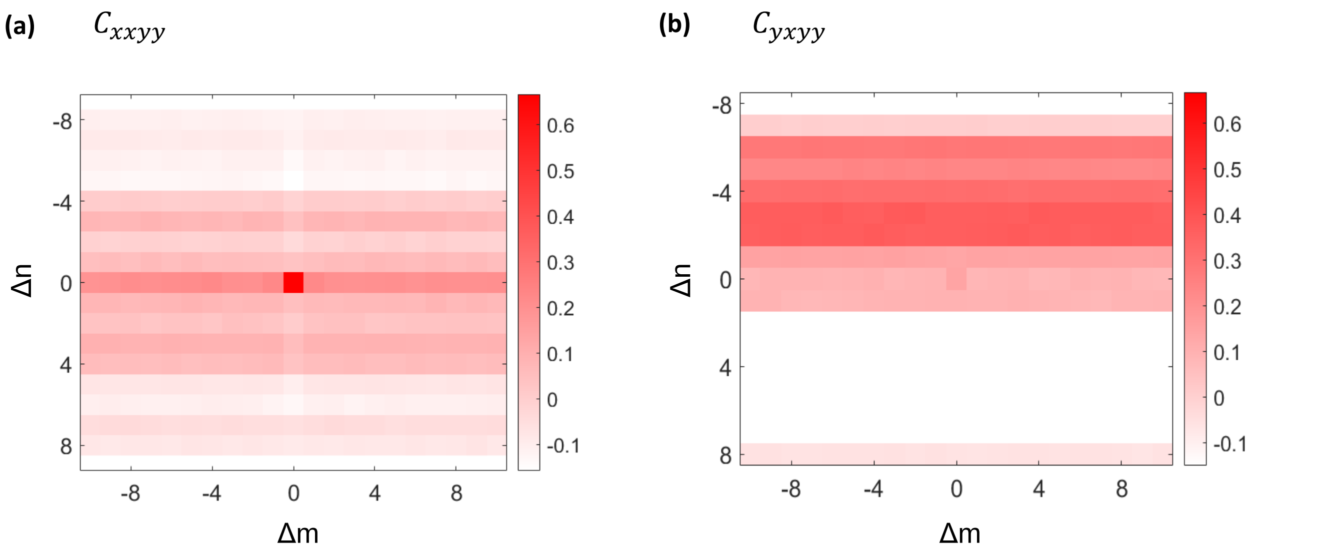

Supplementary Figure 5. Correlations among the vectorial transmission matrix elements. (a) Cross correlation matrix C_{xxyy} between the diagonal sub-matrices t^{xx} and t^{yy} and (b) between the off-diagonal t^{yx} and t^{yy} (C_{xxyy}).

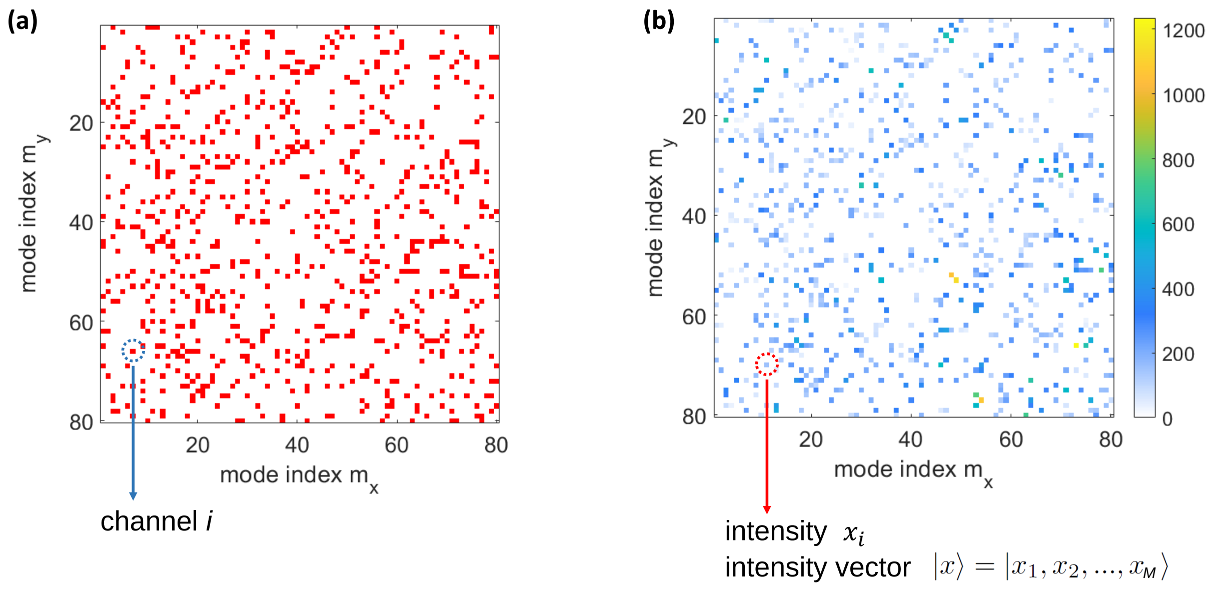

Supplementary Figure 6. Selection of the output channels. (a) A set of $M = 800$ randomly-positioned channels used for single-shot polarimetry of vector beams. Red points mark open (selected) channels. (b) The intensity distribution on the channel set gives the collected data.

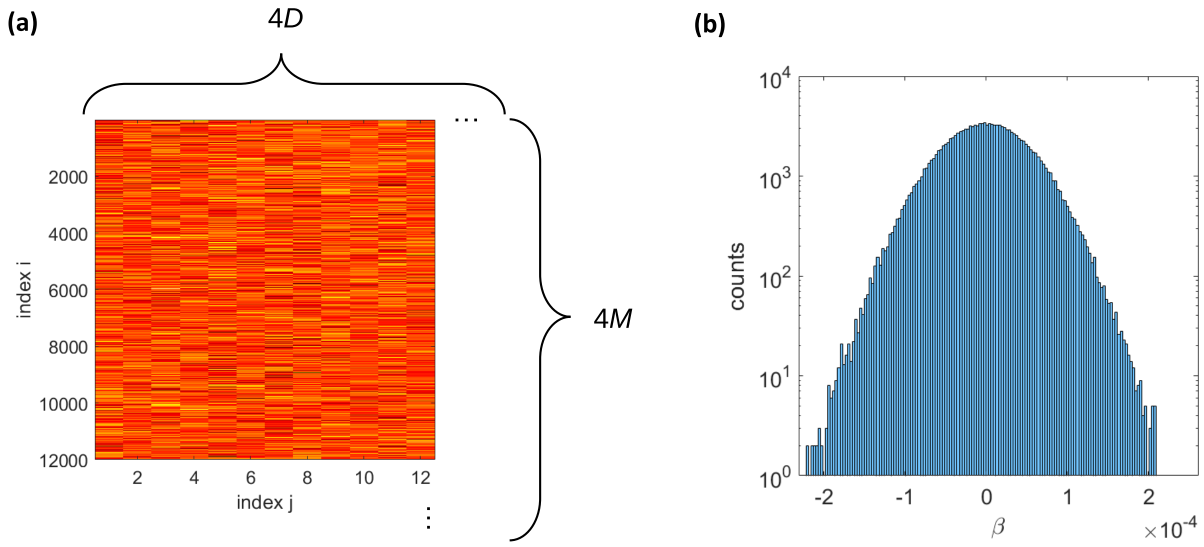

Supplementary Figure 7. Calibration matrix. (a) Part of a $4D \times 4M$ calibration matrix when trained on multiple SOP with $D = 4$. (b) Histogram counts of the β_i^j values in (a).

Supplementary References

[1] B.Schaefer, E.Collett, R.Smyth, D.Barrett, B.Frahar, Measuring the Stokes polarization parameters, *Am. J. Phys.* **75**, 2 (2007).

[2] S.M. Popoff, G. Lerosey, R. Carminati, M. Fink, A.C. Boccara, and S. Gigan, Measuring the Transmission Matrix in Optics: An Approach to the Study and Control of Light Propagation in Disordered Media *Phys. Rev. Lett.* **104**, 100601 (2010).

[3] S. Tripathi, R. Paxman, T. Bifano, K. C. Toussaint, Vector transmission matrix for the polarization behavior of light propagation in highly scattering media, *Opt. Express***20**, 16067–16076 (2012).

[4] Y. Guan, O. Katz, E. Small, J. Zhou, and Y. Silberberg, Polarization control of multiply scattered light through random media by wavefront shaping, *Opt. Lett.* **37**, 4663 (2012).

[5] H.B. de Aguiar, S. Gigan, S. Brasselet, Polarization recovery through scattering media, *Sci. Adv.* **3**, e1600743 (2017).

[6] D. Pierangeli, G. Marcucci, and C. Conti, Photonic extreme learning machine by free-space optical propagation, *Photonics Res.* **9** 1446-1454 (2021).

[7] A. Boniface, J. Dong, and S. Gigan, Non-invasive focusing and imaging in scattering media with a fluorescence-based transmission matrix, *Nat. Commun.* **11**, 6154 (2020).
